# Antimicrobial Susceptibility Profile and Whole-Genome Analysis of a Strong Biofilm-Forming *Bacillus* Sp. B87 Strain Isolated from Food

**DOI:** 10.3390/microorganisms10020252

**Published:** 2022-01-23

**Authors:** Phornphan Sornchuer, Kritsakorn Saninjuk, Parisa Prathaphan, Rattana Tiengtip, Suphot Wattanaphansak

**Affiliations:** 1 Microbiology and Immunology, Department of Preclinical Science, Faculty of Medicine, Thammasat University, Klongluang 12120, Pathum Thani, Thailand; 2 Thammasat University Research Unit in Nutraceuticals and Food Safety, Faculty of Medicine, Thammasat University, Klongluang 12120, Pathum Thani, Thailand; parisaprathaphan@gmail.com; 3 Departments of Veterinary Medicine, Faculty of Veterinary Science, Chulalongkorn University, Bangkok 10330, Thailand; kritsakorn.san@gmail.com (K.S.); supot.W@chula.ac.th (S.W.); 4 Laboratory Section, Faculty of Medicine, Thammasat University, Klongluang 12120, Pathum Thani, Thailand; rattana-nou@hotmail.com

**Keywords:** *Bacillus* sp., whole-genome sequencing, biofilm, virulence factors, antimicrobial resistance

## Abstract

Members of the *Bacillus cereus* group are considered to be foodborne pathogens commonly associated with diarrheal and emetic gastrointestinal syndromes. Biofilm formation is a major virulence determinant of various pathogenic bacteria, including the *B. cereus* strains, since it can protect the bacteria against antimicrobial agents and the host immune response. Moreover, a biofilm allows the exchange of genetic material, such as antimicrobial resistance genes, among the different bacterial strains inside the matrix. The aim of the current study was to genotypically and phenotypically characterize *Bacillus* sp. B87, a strain that was isolated from food and which exhibited strong biofilm-forming capacity. Based on the analysis of the phylogenetic relationship, the isolate was phylogenetically mapped close to *Bacillus pacificus*. Antimicrobial susceptibility testing revealed that the isolate was resistant to tetracycline and β-lactam antimicrobial agents, which corresponded with the genotypic characterization using the whole-genome analysis. The genome of *Bacillus* sp. B87 carried the three-component non-hemolytic enterotoxin (NHE), which is a type of enterotoxin that causes diarrheal symptoms. In addition, the genome also contained several genes that participate in biofilm formation, including the *pelDEA_DA_FG* operon. These findings expand our understanding of antimicrobial resistance and virulence in *Bacillus* species based on the link between genotypic and phenotypic characterization.

## 1. Introduction

*Bacillus cereus* are Gram-positive, spore-forming bacteria that inhabit food, soil, and other natural media. *B. cereus* is a known human pathogen that causes food poisoning with emetic or diarrheal symptoms. Emetic strains of *B. cereus* can secrete the highly thermo- and pH-resistant toxin cereulide, which is pre-formed in food and causes vomiting a few hours after consumption [1]. Cereulide is an ionophoric dodecadepsipeptide that is produced by cereulide synthetase or Ces non-ribosomal peptide synthetase. For diarrheal strains of *B. cereus*, spores in contaminated food are consumed by the host, germinate within the small intestine, and the resulting vegetative cells then grow and produce enterotoxins. Three types of enterotoxins are secreted by *B. cereus*: the three-component enterotoxin hemolysin BL (HBL), the three-component non-hemolytic enterotoxin (NHE), and the single-component enterotoxin cytotoxin K (CytK) [2]. In addition to enterotoxins, *B. cereus* produces several other toxins and degradative enzymes, with most of these products controlled by the PlcR transcriptional activator [3]. PlcR is one of the *B. cereus* quorum-sensing systems that helps the bacterium adapt to diverse conditions [4]. 

*B. cereus* is typically resistant to β-lactam antimicrobial agents, such as penicillin G, ampicillin, and cefotaxime [5], due to the production of β-lactamase enzymes [6]. Bacterial resistance to commonly used antimicrobial agents, such as erythromycin, tetracycline, and streptomycin, can be a consequence of both nature and nurture [5,7]. Tetracycline is a broad-spectrum antimicrobial agent with activity against a wide range of bacteria, including Gram-positive and Gram-negative isolates. *B. cereus* is generally susceptible to tetracycline, but the resistance of *B. cereus* to this antimicrobial agent has been reported in some countries [8].

Biofilm formation is a major virulence determinant of various pathogenic bacteria, especially in the *B. cereus* group [9]. The formation of biofilms by bacteria can be associated with chronic infections in human and animal hosts. Moreover, biofilm formation allows the development and transfer of antimicrobial resistance through the bacterial interactions that occur within the biofilm [10,11]. The key genes for biofilm formation comprise those encoding biofilm transcriptional regulators, matrix structural genes, potential extracellular DNA synthesis genes, and cyclic-di-GMP metabolism genes [12]. In addition, several gene loci are involved in biofilm formation, including genes encoding the lipopeptide kurstakin, genes encoding the cyclic-di-GMP responsive effector protein BspA, and genes encoding the c-di-GMP synthesizing enzyme [13,14,15,16]. However, there may be strain-dependent variation in the mechanisms of biofilm formation among members of the *B. cereus* group. 

The accessibility of whole-genome sequencing (WGS) has facilitated the assessment of bacterial genomes through bioinformatics analysis for the genetic potential to produce virulence factors and proteins involved in antimicrobial resistance and biofilm formation. In this study, the strong biofilm-forming strain, *Bacillus* sp. B87, isolated from food, was characterized genotypically and phenotypically, and genomic comparisons with other relevant *B. cereus* genomes were performed. The study aimed to generate insights into the genetic basis of antimicrobial resistance and virulence of this foodborne pathogen.

## 2. Materials and Methods

### 2.1. Bacterial Strains and Culture Conditions

The *Bacillus* sp. B87 used in this study was isolated from a spicy mussel salad in Pathum Thani province, Thailand [17]. Bacteria were aerobically grown in Luria–Bertani (LB) broth (Difco Laboratories, Detroit, MI, USA), with shaking at 180 rpm at 35 ± 2 °C.

### 2.2. Biofilm Formation Assay

The biofilm formation assay was based on a previously described protocol with minor modifications [18]. Briefly, *Bacillus* sp. B87 was grown overnight in LB broth at 35 °C and 180 rpm to generate inoculum cultures. Overnight cultures were adjusted to an optical density at 600 nm (OD_600_) of 0.01 in LB. Next, 100 µL of the adjusted bacterial culture was added to each well of a pre-sterilized 96-well flat-bottomed polystyrene microtiter plate, followed by incubation at 35 °C and 50 rpm for 24 h. Planktonic bacteria were then removed, and the wells were washed with distilled water and air-dried. Biofilm cells were stained with 200 µL of 0.3% crystal violet for 10 min, washed with distilled water, and air-dried. The crystal violet in the biofilm cells was solubilized with 200 µL of 70% ethanol, and the optical density at 590 nm (OD_590_) was measured.

### 2.3. Antimicrobial Susceptibility Tests

The antimicrobial susceptibility of *Bacillus* sp. B87 was determined using the Kirby–Bauer disk diffusion method according to standard criteria of the Clinical and Laboratory Standards Institute (CLSI) 2010 [19]. Briefly, the isolate was grown overnight at 35 °C on a nutrient agar (NA; Oxoid, United Kingdom) and the culture was compared with 0.5 McFarland turbidity standards. The culture was then applied onto Mueller–Hinton agar (MHA) plates using a sterile cotton swab, and the inoculated plates were dried at room temperature. The antimicrobial agents tested in this study included ampicillin (AMP, 10 µg), amoxicillin–clavulanic acid (AMC, 20 µg/10 mg), penicillin G (PEN, 10 U), gentamicin (GEN, 10 µg), imipenem (IPM, 10 µg), vancomycin (VAN, 30 µg), chloramphenicol (CHL, 30 µg), ciprofloxacin (CIP, 5 µg), tetracycline (TET, 30 µg), trimethoprim–sulfamethoxazole (SXT, 1.25 µg/23.75 µg), and erythromycin (ERY, 15 µg). Based on the zones of inhibition, *Bacillus* sp. B87 was classified as sensitive (S), intermediate (I), or resistant (R) to each antimicrobial agent according to the interpretative criteria for *Staphylococcus* spp., following CLSI guidelines [20]. *Staphylococcus aureus* ATCC 25923 was used as a control strain for the antimicrobial susceptibility tests. 

### 2.4. Whole-Genome Sequencing, Assembly, and Annotation

Genomic DNA was extracted from *Bacillus* sp. B87 using a GF-1 Bacterial DNA Extraction Kit (Vivantis Technologies, Selangor, Malaysia) according to the manufacturer’s instructions. DNA quality was assessed via spectrophotometry and gel electrophoresis. Purified high molecular weight DNA samples with a 260/280 nm absorbance ratio of 1.8–2.0 and a 260/230 nm absorbance ratio of 2.0–2.2 were used for library construction and sequencing. The DNA sequencing library was prepared using a QIAGEN FX kit (Qiagen, Valencia, CA, USA), which fragments the gDNA using an enzymatic reaction, cleans the fragmented DNA with magnetic beads, and then ligates an adaptor index to the fragmented DNA. The quality and quantity of the indexed libraries were determined using an Agilent 2100 Bioanalyzer and a Denovix fluorometer, and the libraries were then pooled in equimolar quantities. Cluster generation and paired-end 2 × 150 nucleotide read sequencing were performed on an Illumina HiseqXten (Illumina Inc., San Diego, CA, USA). 

The quality of the raw sequencing reads was assessed using FASTQC software. Adaptors and poor-quality reads were removed using Fastp, and the filtered reads were used as inputs for the Unicycler genome assembly program. The genome of *Bacillus* sp. B87 was annotated with the Rapid Annotation using Subsystem Technology tool kit (RASTtk) in PATRIC (Pathosystems Resource Integration Center). Sequences were queried using the BTyper tool, the Virulence Factor Database (VFDB) and Victors resource (for virulence factors), and the Comprehensive Antibiotic Resistance Database (CARD) and the NCBI National Database of Antibiotic Resistant Organisms (NDARO) (for antimicrobial resistance). In addition, the functional annotation of genes in terms of the Kyoto Encyclopedia of Genes and Genomes (KEGG) orthology assignments and predictions of KEGG pathways were performed through the KEGG Automatic Annotation Server (KAAS; https://www.genome.jp/kegg/kaas/ accessed on 18 October 2021) using the bi-directional best hit (BBH) method [21]. The circular genome map was constructed by using the circular viewer of PATRIC. A comparison of syntenic analyses in *Bacillus* sp. B87 and other species of the genus *Bacillus* was performed by using the Easyfig program [22].

### 2.5. Phylogenetic Analysis

A phylogenomic tree based on the core genes was generated with PATRIC Phylogenetic Tree Building Service [23]. The default was set for codon trees, which utilizes both the protein and gene sequences from PATRIC’s global protein families (PGFams). Protein sequences were aligned using MUSCLE, and the nucleotide coding gene sequences were aligned using the Codon_align function of BioPython. A concatenated alignment of all proteins and nucleotides was converted to a phylip formatted file, and then a partitions file for RaxML was constructed. Support values were created using 100 rounds of the “Rapid” bootstrapping option of RaxML. The phylogenomic classification of Bacillus sp. B87 was also performed using the Type (strain) Genome Server (TYGS), a free bioinformatics platform for a whole-genome-based taxonomic analysis [24]. 

### 2.6. Nucleotide Sequence Accession Numbers

The draft genomes of *Bacillus* sp. B87 were deposited in the NCBI database under the accession numbers SRR16129018. The BioProject ID in GenBank is PRJNA7260213. 

## 3. Results

### 3.1. Biofilm-Forming Ability of Bacillus Sp. B87

**Figure 1:**
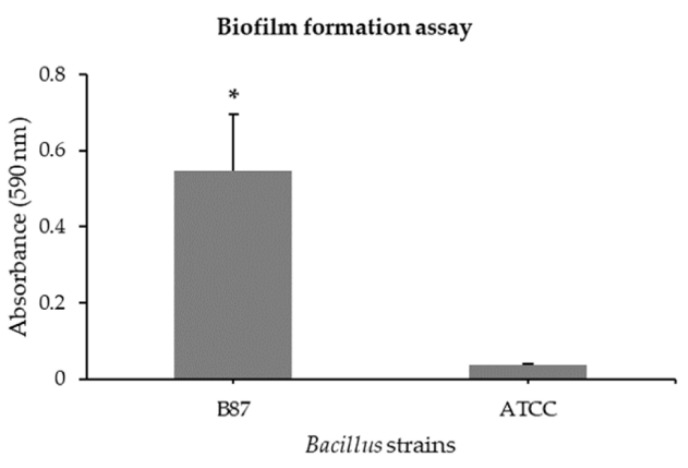
Biofilm formation assay of *Bacillus* sp. B87. Submerged biofilms of *Bacillus* sp. B87 and *B. cereus* ATCC 14579 were visualized via crystal violet staining. Data represent the mean of three independent experiments, each consisting of six internal replicates. Error bars indicate standard deviations. Statistical analysis includes Student’s paired *t*-test (* *p* < 0.01).

The ability of *Bacillus* sp. B87 to produce biofilm was determined in comparison with *B. cereus* ATCC 14579. A microtiter plate assay for biofilm formation revealed that the biofilm level of *Bacillus* sp. B87 (OD_590_ 0.545 ± 0.149) after incubation for 24 h was significantly higher than that of *B. cereus* ATCC 14579 (OD_590_ 0.035 ± 0.002) (Figure 1).

### 3.2. Antimicrobial Resistance Profile of Bacillus Sp. B87

*Bacillus* sp. B87 was tested for susceptibility to 11 selected antimicrobial agents, as shown in Table 1. *Bacillus* sp. B87 was susceptible to most of the tested antimicrobial agents, including GEN (24.8 mm. ± 1.4), IPM (39.4 mm. ± 0.3), VAN (22.8 mm. ± 0.5), CHL (30.7 mm. ± 0.7), CIP (33.2 mm. ± 1.7), SXT (21.0 mm. ± 1.6), and ERY (28.8 mm. ± 0.6). The isolate was resistant to antimicrobial agents in the β-lactam category, including AMP (14.3 mm. ± 0.8), AMC (8.2 mm. ± 0.7), and PEN (10.6 mm. ± 2.3), and was also resistant to tetracycline (12.5 mm. ± 1.4).

### 3.3. Genetic Features of Bacillus Sp. B87

**Figure 2:**
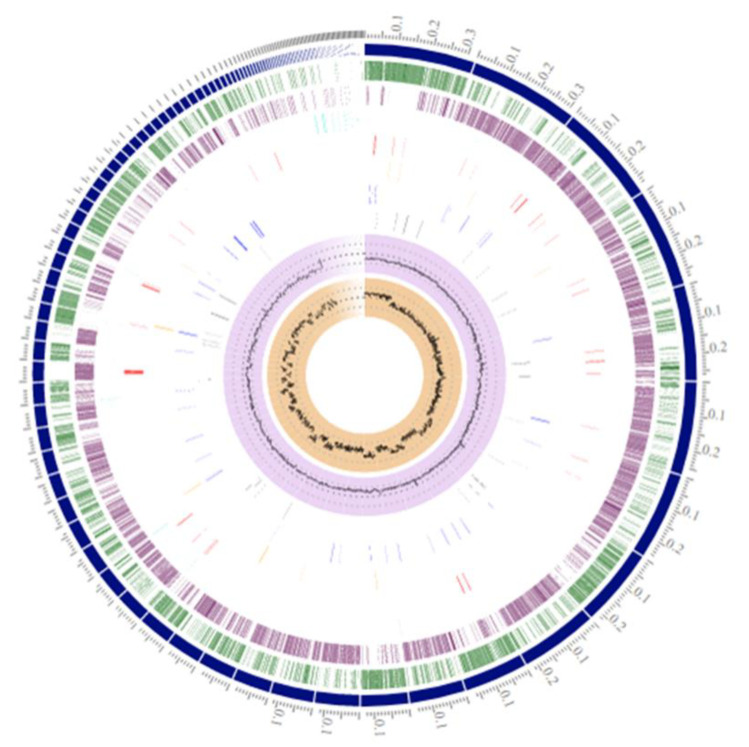
Circular representation of the *Bacillus* sp. B87 draft genome. Circular genome visualization was generated using the circular viewer of PATRIC. Outer to center: contigs, forward CDS, reverse CDS, non-CDS features, AMR genes, VF Genes, transporters, and drug targets. The two inner tracks are GC content and GC skew.

**Figure 3:**
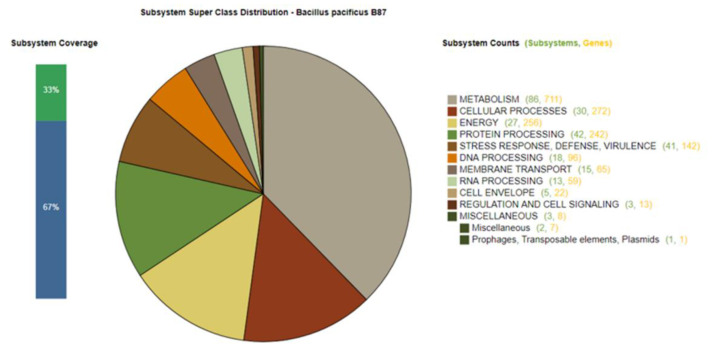
Overview of the subsystem categories of the annotated draft whole genome of *Bacillus* sp. B87 from the RAST server. The pie chart shows the number of genes related to individual subsystems. The bar graph on the left reveals the subsystem coverage. The ratio of coding sequences annotated in the SEED subsystem (33%) and outside of the SEED subsystem (67%) is indicated.

Genomic features and annotation information for the genome of *Bacillus* sp. B87 are summarized in Table 2. The draft genome sequence had an estimated length of 5,448,163 bp, a GC content of 35.18%, and contained 5661 coding sequences. The circular representation of the *Bacillus* sp. B87 draft genome was generated using the circular viewer of PATRIC and is shown in Figure 2. Potential genes in the draft genome of *Bacillus* sp. B87 were investigated and annotated based on different biological processes and metabolic pathways using the RAST server (Figure 3). The predicted genes included 711 genes involved in metabolism, 272 genes involved in cellular processes, and 142 genes involved in stress response, defense, and virulence. Genes involved in prophages, transposable elements, and plasmids were also found in the draft genome of *Bacillus* sp. B87, and were classified as: subclass: pathogenicity islands; subsystem name: Listeria Pathogenicity Island LIPI-1 extended. These pathogenicity islands were also present in the genomes of *B. cereus* ATCC 14579 and *B. anthracis* str. Ames. 

**Figure 4:**
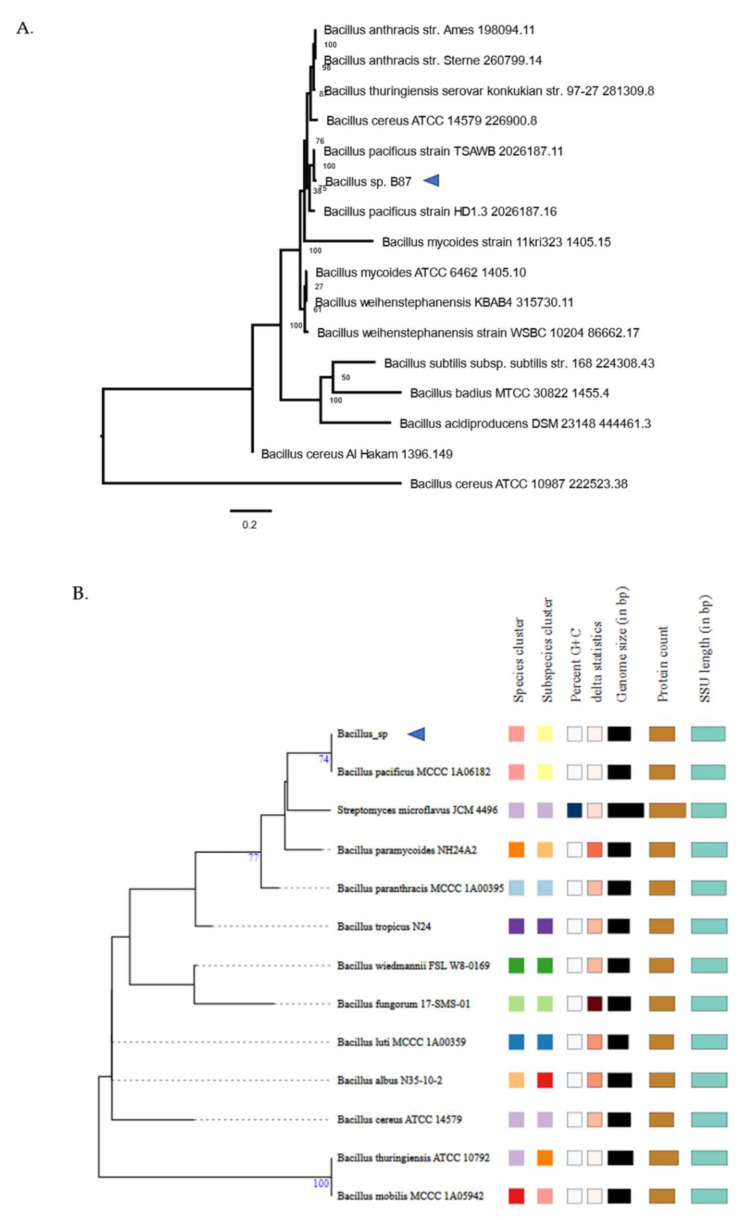

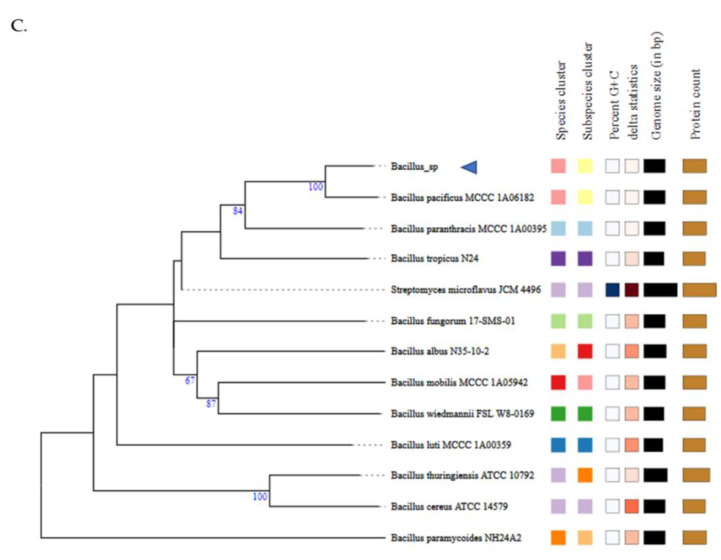
Phylogenetic analysis of *Bacillus* sp. B87. (**A**) The codon tree method selects single-copy PATRIC PGFams and analyzes aligned proteins and coding DNA from single-copy genes using the program RAxML. (**B**,**C**) TYGS results of *Bacillus* sp. B87 based on 16S *rRNA* gene (**B**) and whole-genome (**C**) sequences.

A phylogenetic tree based on core genes was reconstructed in PATRIC using the whole-genome sequence of *Bacillus* sp. B87 (Figure 4A). This phylogenomic analysis revealed that *Bacillus* sp. B87 was closely related to strains of *Bacillus pacificus*. The 16S *rRNA* gene (Figure 4B) and whole-genome (Figure 4C) phylogeny reconstructions using TYGS confirmed a close association of *Bacillus* sp. B87 with *B. pacificus*.

### 3.4. Antimicrobial Resistance Genes

Antimicrobial resistance (AMR) genes that are associated with resistance to one or more antimicrobial agents were predicted based on the CARD and NDARO databases, and *Bacillus* sp. B87 contained nine genes connected with resistance to different antimicrobial agents (Table 3). Moreover, 43 AMR genes were annotated according to the PATRIC database using K-mer Search (Supplementary Table S1). These findings might be associated with the observed antimicrobial resistance phenotypes to β-lactam antimicrobial agents and tetracycline shown in Table 1.

### 3.5. Biofilm Formation Genes

According to RASTtk, available in PATRIC, the genes involved in the formation of biofilm by *Bacillus* sp. B87 were identified. Nine genes were detected (Table 4) according to the following hierarchical classification: superclass: cellular process; class: microbial communities; subclass: quorum sensing and biofilm formation. Key biofilm-formation genes were also investigated using the KAAS database, and this analysis identified the genes encoding biofilm transcriptional regulators, matrix protein-encoding genes, putative matrix polysaccharide synthesis genes, and extracellular DNA (eDNA) synthesis genes (Table 5). 

**Figure 5:**
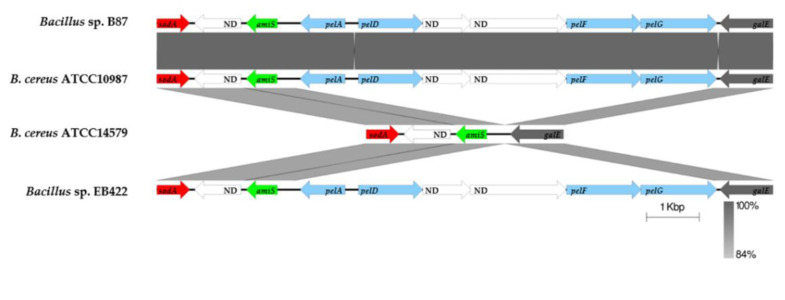
Genetic organization of the *pelDEA_DA_FG* operon. The *pelDEA_DA_FG* operons were compared using the Easyfig tool with homologous clusters found in related species of the genus *Bacillus*. Arrows indicate the transcription direction of each CDS with different colors (red, *sodA*; green, *amiS*; light blue, *pel*; grey, *galE*; white, unknown function (ND)). The shade of gray indicates the degree of nucleotide sequence homology (%) according to BLASTN.

The differences in gene synteny between the genomes of *Bacillus* sp. B87 and *B. cereus* ATCC 14579 were apparent with the *pelDEA_DA_FG* operon (Figure 5). *Bacillus* sp. B87 contained *pelA*, *pelD*, *pelF*, and *pelG*, and was therefore similar to *B. cereus* ATCC 10987 and *Bacillus* sp. EB422 (*B. pacificus*).

### 3.6. Virulence Factor Genes

Thirteen genes in the draft genome of *Bacillus* sp. B87 were classified as virulence factors according to the Victors (nine genes) and VFDB (four genes) databases (Table 6). Among these genes were *nheA*, *nheB*, and *nheC*, which encode the three-component NHE complex, a type of enterotoxin that causes diarrheal symptoms. However, the cytotoxin K virulence factor, which is also associated with diarrheal illness and was present in *B. cereus* ATCC 14579, was not detected in *Bacillus* sp. B87 (Supplementary Table S2). Virulence genes were also predicted by using the BTyper tool, and this analysis predicted 17 virulence genes in the genome of *Bacillus* sp. B87 (Table 6).

## 4. Discussion 

This study aimed to genotypically and phenotypically characterize *Bacillus* sp. B87, a strain with strong biofilm-forming activity that was previously isolated from a spicy mussel salad in Pathum Thani province, Thailand. The phylogenetic analysis revealed that *Bacillus* sp. B87 was closely related to *B. pacificus*, which had been reported to be isolated from the sediment of the Pacific Ocean [25]. Currently, little is known about the phenotype and genotype of this bacterial strain.

The alignment of the *Bacillus* sp. B87 genome indicated that the isolate possesses the genes *nheA*, *nheB*, and *nheC*, but not *hblA*, *hblC*, *hblD*, or *cytK*. In *B. cereus*, the toxins that are associated with diarrheal diseases are HBL, NHE, CytK, and enterotoxin FM [26,27,28,29,30,31,32]. Indeed, isolates that carry the genes encoding *HBL* might be more virulent [33]. However, a strain lacking the *HBL* operon—*B. cereus* ATCC 10987—was reported to exhibit strong cytopathogenic activity in Vero cells, since it could produce a large amount of the *NHE* mRNA [34]. The genome of *Bacillus* sp. B87 also contains Listeria Pathogenicity Island LIPI-1 extended. LIP-1 in *Listeria monocytogenes* harbors several important genes, including *prfA*, *plcA*, *hly*, *mpl*, *actA*, and *plcB*, which are involved in host invasion and cellular proliferation [35]. Orthologs for LIP-1 were also detected in *B. cereus* ATCC 14579 and *B. cereus* strain FORC_021, which was isolated from a knife used at a sashimi restaurant in the Republic of Korea [36]. However, the coding sequence (CDS) counts of LIPI-1 genes in each strain were dissimilar in that report. The presence of Listeria Pathogenicity Island LIPI-1 extended in *Bacillus* sp. B87 might be responsible for the virulence of this strain and requires further investigation.

*B. cereus* is typically resistant to β-lactam antimicrobial agents, including penicillin G, ampicillin, and amoxicillin–clavulanic acid [17,20,37,38]. Some bacterial strains can produce β-lactamase enzymes that are responsible for the resistance to β-lactam antimicrobial agents. Three different β-lactamases have been classified in strains of *B. cereus*, namely, β-lactamase I, II, and III [39]. *B. cereus* β-lactamase II (BcII) is a heat-stable metallo-β-lactamase (MBL) that shares high sequence homology with Bla2 from *B. anthracis* [40]. MBL catalyzes the hydrolysis of β-lactam antimicrobial agents, including penicillin, cephalosporin, carbapenem, and cephamycin [41]. However, BcII catalyzes the hydrolysis of penicillin at higher rates than cephalosporin and carbapenem [42]. The current study revealed the presence of the *BcII* gene in the draft genome of *Bacillus* sp. B87, and this gene might play an important role in the β-lactam resistance phenotype of the isolate.

*Bacillus* sp. B87 carries the *tet*(45) gene, which contributes to the tetracycline resistance phenotype in this isolate. Tet45 is a tetracycline efflux pump closely related to TetL [43]. Homologs of Tet45 have been found in the genomes of strains of *B. cereus* and *B. thuringiensis*. Furthermore, *tetA*-carrying *B. cereus* was reported to be susceptible to tetracycline [44]. Moreover, some isolates of *B. cereus* were phenotypically resistant to tetracycline, even though they did not carry *tetA*, *tetB*, or *tetC*, and this might be due to the presence of other tetracycline resistance genes, such as *tetM* and *tetL* [45]. *B. cereus* is generally susceptible to tetracycline, but the resistance of *B. cereus* to tetracycline has been reported in some countries [8]. In the current study, *Bacillus* sp. B87 was resistant to tetracycline, since it possessed the gene encoding the tetracycline resistance efflux pump. This phenotype is concordant with a previous report on *B. cereus* strain MS532a, which presented a tetracycline-resistant phenotype and carried the *tet(45)* tetracycline resistance gene [38]. Horizontal gene transfer may have contributed to the dissemination and persistence of *tet(45)* in environments such as a poultry litter-impacted soil [43]. The presence of the *tet(45)* gene in the genome of *Bacillus* sp. B87, a strain isolated from spicy mussel salad, may be due to the contamination of vegetables, mussels, or other ingredients. Tetracycline-resistant isolates of *B. cereus* have previously been shown to carry the *tet(L)* gene on a plasmid, while other species of the genus *Bacillus* carry either *tet(L)* or *tet(K)* on plasmids and/or in the chromosome [46,47,48]. Both genes—*tet(L)* and *tet(K)*—can occasionally be mobilized in the presence of conjugative plasmids, but are not themselves able to independently transfer, thus decelerating their spread within the population [45]. The ability to form biofilm in bacteria is an important mechanism that allows the bacteria to resist antimicrobial agents and disinfectants, as well as to evade the host immune system. *B. cereus* can form biofilms on various surfaces [49,50,51], including plastic, glass wool, and stainless steel, and the biofilm cells were more resistant to sanitizers compared with planktonic cells [52]. Biofilm plays an important role in the exchange of antimicrobial resistance genes [53]. In strains of *Escherichia coli* and *Pseudomonas aeruginosa*, sub-inhibitory concentrations of tetracycline and cephradine induce biofilm formation and increase the transfer rate of the pB10 plasmid among the biofilm biomass at higher rates compared with no antimicrobial treatment [54]. The findings from the current study were congruent with a previous report [55] that resistant bacteria could form biofilms. Tetracycline-resistant *Bacillus* sp. B87, with strong biofilm-formation ability, may develop greater resistance to different antimicrobial agents and other environmental stressors if left unmonitored.

The regulation network controlling *B. cereus* biofilm formation involves several pathways. PlcR is a pleiotropic regulator that controls the expression of genes encoding several enterotoxins, hemolysins, phospholipases, and proteases in *B. cereus* [9,56]. The *plcR* gene is instrumental in biofilm formation, since the deletion of this gene in *B. cereus* ATCC 14579 resulted in a significant increase in the amount of biofilm [57]. Two roles have been reported for the regulator CodY: the repression of biofilm formation in *B. cereus* ATCC 14579 [58] and the promotion of biofilm formation in *B. cereus* UW101C [59]. The *codY* gene operon has a role in pellicle biofilm formation and swarming motility [60]. An operon including *tasA*, *tapA*, and *sipW* has been proposed to be involved in biofilm formation in *B. subtilis* [61]. The transcription of these genes is regulated by SinI and repressed by SinR [62]. The transcription of *sinI* is activated by the master regulator of sporulation Spo0A [63]. In the present study, *Bacillus* sp. B87 was found to carry the genes *tasA* and *sipW*, which may be sufficient for biofilm production. The deletion of the genomic region encoding two orthologs of the amyloid-like protein TasA and SipW signal peptidase inhibited biofilm assembly [64]. However, the genes *calY* and *tapA* were not detected in the isolate. Mutations in *tasA* or *calY* did not completely prevent biofilm formation. Moreover, the lack of *tapA* may not completely interrupt biofilm production, which was congruent with a previous report [65].

Several gene loci are involved in biofilm formation, and there may be strain-to-strain variability in matrix component utilization among isolates of *B. cereus*. For example, the *pelDEA_DA_FG* operon identified in strain ATCC 10987 is a crucial locus for biofilm formation in *B. cereus*. However, this locus is not present in strain ATCC 14579 of this species. This operon might be required for biofilm formation in *Bacillus cereus* strains, at least on polystyrene surfaces, since ATCC 10987 formed biofilms, while ATCC 14579 did not form biofilms on polystyrene 96-well plates [66]. In addition, the deletion of any of *pelDEA_DA_FG* in ATCC 10987 resulted in the reduction of bacterial adhesion to the wells of the plastic microtiter dish [67]. The bioinformatics analysis and validation of Pel production in *Bacillus* sp. B87 in the current study suggested that the *pelDEA_DA_FG* operon was present and potentially functional in biofilm-forming strains. In addition, the presence of the gene *recA* in the genome of *Bacillus* sp. B87 may enhance the biofilm-forming capacity of this isolate, since it has been reported that the biofilm formation and swarming motility of *B. cereus* 905 are promoted by RecA [68]. However, further investigation via a gene deletion approach is required to confirm this theory.

## 5. Conclusions 

This study reported the phenotypic and genotypic characterization of *Bacillus* sp. B87, an isolate with strong biofilm-forming capacity. The isolate was resistant to β-lactam antimicrobial agents and tetracycline, since it carried *BcII* and *tet(45)* genes in the genome. Consequently, the strain can survive in tetracycline-containing environments where it may induce the biofilm-forming capacity of this isolate, and this assumption needs to be further elucidated. Moreover, the presence of the *pelDEA_DA_FG* operon in the genome of *Bacillus* sp. B87 might play an important role in the biofilm-forming capacity of this isolate. Biofilm formation would allow the bacteria to exchange genetic material among strains, and could potentially lead to the development of greater resistance to different antimicrobial agents and other environmental stressors. In conclusion, the identification of genes encoding virulence factors and antimicrobial resistance in foodborne bacteria should be considered to be potential key points to assess human health risks from the bacteria. Moreover, the findings from this study suggest that WGS analysis could be an effective tool to elucidate the pathogenic potential of the *B. cereus* group. However, the molecular basis proposed in this study needs to be further clarified through gene knockout and protein characterization, since several gene homologs in members of the *B. cereus* group have unique and varied functions. 

## Figures and Tables

**Table 1:** Antimicrobial susceptibility testing of *Bacillus* sp. B87.

Category	Antimicrobial Agent	Interpretation
β-lactam	Ampicillin (10 µg)	R
	Amoxicillin–clavulanic acid (20 µg/10 µg)	R
	Penicillin (10 U)	R
Aminoglycosides	Gentamicin (10 µg)	S
Carbapenems	Imipenem (10 µg)	S
Glycopeptides	Vancomycin (30 µg)	S
Phenicols	Chloramphenicol (30 µg)	S
Fluoroquinolones	Ciprofloxacin (5 µg)	S
Tetracyclines	Tetracycline (30 µg)	R
Folate pathway inhibitors	Trimethoprim–sulfamethoxazole (1.25 µg/23.75 µg)	S
Macrolides	Erythromycin (15 µg)	S

S: Sensitive, R: Resistant.

**Table 2:** Genomic features and annotation information of the chromosome of *Bacillus* sp. B87.

Genome Features	Chromosome
Genome length (bp)	5,448,163
Protein-coding genes	5661
GC content (%)	35.18
The number of tRNA	77
The number of rRNA	5
Contigs	117
Contig L50	11
Contig N50	161,893

**Table 3:** AMR genes prediction of *Bacillus* sp. B87 based on NDARO and CARD databases.

Genes	Product	Source ID	Source Organism
CARD database			
	Translation elongation factor Tu	YP_006374661.1	*Enterococcus faecium* DO
*BLA1*	Class A beta-lactamase (EC 3.5.2.6)	AAR20595.1	*B. anthracis*
*FosB*	Fosfomycin resistance protein FosB	NP_831795.1	*B. cereus* ATCC 14579
*BcII*	Subclass B1 beta-lactamase (EC 3.5.2.6) => BcII family	AAA22562.1	*B. cereus*
*dfrE*	Thymidylate synthase (EC 2.1.1.45)	AAD01867.1	*E. faecalis*
NDARO database			
	Class A beta-lactamase (EC 3.5.2.6)	WP_063842248.1	*B. cereus*
	Subclass B1 beta-lactamase (EC 3.5.2.6) => BcII family	WP_000799223.1	*B. cereus* group
	Fosfomycin resistance protein FosB	WP_000943763.1	*Bacillus*
	Tetracycline resistance, MFS efflux pump => Tet(45)	WP_063855885.1	*Bhargavaea cecembensis*

**Table 4:** Prediction of biofilm formation genes of *Bacillus* sp. B87 based on RASTtk in PATRIC.

Biofilm Formation Genes	Product
*lsrR*	Transcriptional repressor of *lsr* operon
*lsrK*	Autoinducer 2 (AI-2) kinase LsrK (EC 2.7.1.-)
*lsrD*	Autoinducer 2 (AI-2) ABC transport system, membrane channel protein LsrD
*lsrC*	Autoinducer 2 (AI-2) ABC transport system, membrane channel protein LsrC
	Cupin domain protein in autoinducer 2 (AI-2)-related operon
	N-acyl homoserine lactone hydrolase
	3-hydroxy-5-phosphonooxypentane-2,4-dione thiolase (EC 2.3.1.245)
	Autoinducer 2 (AI-2) ABC transporter, dimeric ATP-binding protein
	Autoinducer 2 (AI-2) ABC transporter, substrate-binding protein

**Table 5:** Identification of genes in *Bacillus* sp. B87 relevant to biofilm formation annotated using the KAAS database.

KEGG Orthology	Genes	Protein Product
Biofilm transcriptional regulators		
K06284	*abrB*	AbrB family transcriptional regulator, transcriptional pleiotropic regulator of transition state genes
K03706	*codY*	Transcriptional pleiotropic repressor
K20480	*nprR*	HTH-type transcriptional regulator, quorum-sensing regulator NprR
K20391	*plcR*	HTH-type transcriptional regulator
K20390	*papR*	Regulatory peptide PapR
K06372	*sinI*	Antagonist of SinR
K19449	*sinR*	XRE family transcriptional regulator, master regulator for biofilm formation
K07699	*Spo0A*	Two-component system, response regulator, stage 0 sporulation protein A
Matrix protein-encoding genes		
K06336	*tasA*	Spore coat-associated protein N
K13280	*sipW*	Signal peptidase I
Putative matrix polysaccharide synthesis genes		
K07705	*lytR*	Two-component system, LytTR family, response regulator LytT
K00012	*ugd*	UDPglucose 6-dehydrogenase
K21006	*pelA*	Polysaccharide biosynthesis protein PelA
K21009	*pelD*	Polysaccharide biosynthesis protein PelD
K21011	*pelF*	Polysaccharide biosynthesis protein PelF
K21012	*pelG*	Polysaccharide biosynthesis protein PelG
eDNA synthesis genes		
K01939	*purA*	Adenylosuccinate synthase
K01923	*purC*	Phosphoribosylaminoimidazole-succinocarboxamide synthase
K23269	*purL*	Phosphoribosylformylglycinamidine synthase subunit PurL

**Table 6:** Prediction of genes related to virulence factor of *Bacillus* sp. B87 according to Victors and VFDB databases, and BTyper tool.

Genes	Product	Source ID	Source Organism
Victors database			
*sodA2*	Superoxide dismutase [Mn] (EC 1.15.1.1)	227818216	*B. anthracis* str. CDC 684
*sigB*	RNA polymerase sigma factor SigB	227816152	*B. anthracis* str. CDC 684
*nos*	Nitric oxide synthase oxygenase (EC 1.-.-.-)	227818215	*B. anthracis* str. CDC 684
*codY*	GTP-sensing transcriptional pleiotropic repressor CodY	227813264	*B. anthracis* str. CDC 684
*recA*	RecA protein	15926868	*S. aureus* subsp. *aureus* N315
*phnX*	Phosphonoacetaldehyde hydrolase (EC 3.11.1.1)	47526609	*B. anthracis* str. ‘Ames Ancestor’
*sodC*	Superoxide dismutase [Cu-Zn] precursor (EC 1.15.1.1)	227817676	*B. anthracis* str. CDC 684
*sodA1*	Superoxide dismutase [Mn] (EC 1.15.1.1)	227817051	*B. anthracis* str. CDC 684
*clpX*	ATP-dependent Clp protease ATP-binding subunit ClpX	227817253	*B. anthracis* str. CDC 684
VFDB database			
*nheC*	Enterotoxin C	VFG016286	*B. cereus* ATCC 10987
*inhA*	Immune inhibitor A, metalloprotease (EC 3.4.24.-)	VFG016338	*B. anthracis* str. Sterne
*nheB*	Non-hemolytic enterotoxin lytic component L1	VFG016278	*B. cereus* ATCC 10987
*nheA*	Non-hemolytic enterotoxin A	VFG016270	*B. cereus* ATCC 10987
BTyper tool			
*bpsF*	*Bacillus cereus* exo-polysaccharide operon gene F tyrosine protein kinase [plasmid pBC218]		*B. cereus* str. G9241
*entFM*	Enterotoxin		*B. cereus* ATCC 14579
*bceT*	Diarrheal toxin		*B. cereus*
*plcA*	1-Phosphatidylinositol phosphodiesterase precursor		*B. cereus* ATCC 14579
*entA*	Enterotoxin/cell-wall binding protein		*B. cereus* ATCC 14579
*bpsE*	*Bacillus cereus* exo-polysaccharide operon gene E UTP--glucose-1-phosphate uridylyltransferase [plasmid pBC218]		*B. cereus* str. G9241
*inhA2*	Immune inhibitor A precursor		*B. cereus* ATCC 14579
*nheC*	Enterotoxin C		*B. cereus* ATCC 14579
*cerA*	Cereolysin A		*B. cereus*
*bpsH*	*Bacillus cereus* exo-polysaccharide operon gene H LytR family transcriptional regulator [plasmid pBC218]		*B. cereus* str. G9241
*inhA1*	Immune inhibitor A precursor		*B. cereus* ATCC 14579
*nheA*	Non-hemolytic enterotoxin lytic component L2		*B. cereus* ATCC 14579
*nheB*	Non-hemolytic enterotoxin lytic component L1		*B. cereus* ATCC 14579
*cerB*	Cereolysin B		
*plcB*	Phospholipase C		*B. cereus* ATCC 14579
*sph*	Sphingomyelinase C		*B. anthracis* str. Ames
*plcR*	Transcriptional regulator		*B. anthracis* str. Ames

## Data Availability

The raw sequence generated for this study was deposited into the National Centre for Biotechnology Information (NCBI) Sequence Read Archive (SRA) under the accession numbers SRR16129018, BioProject ID: PRJNA726021. All the other data supporting the finding of this study are available in this published article and its supplementary information files.

## Supplementary Materials

The following are available online at https://www.mdpi.com/article/10.3390/microorganisms10020252/s1, Table S1: PATRIC database using K-mer Search, Table S2: PATRIC virulence factors.
